# Pulsed electron avalanche knife (PEAK) PlasmaBlade™ in pacemaker and defibrillator procedures

**DOI:** 10.1186/s40001-017-0292-7

**Published:** 2017-11-21

**Authors:** Elif Kaya, Matthias Totzeck, Tienush Rassaf

**Affiliations:** Department of Cardiology and Vascular Medicine, West German Heart and Vascular Center Essen, University Hospital Essen, Hufelandstrasse 55, 45147 Essen, Germany

**Keywords:** PEAK PlasmaBlade™, Generator replacement, Implantable cardioverter defibrillator implantation, Pacemaker implantation

## Abstract

**Background:**

The pulsed electron avalanche knife (PEAK) PlasmaBlade™ is an innovative electrosurgical device that uses a novel technology to cut tissues. It has been proven to be safe and feasible in ear, nose, and throat surgery, but there are only limited data concerning the use of PlasmaBlade™ instead of conventional electrocautery in cardiac implantable electronic device (CIED) procedures except for generator replacements.

**Methods:**

We conducted a retrospective, single-center study with patients undergoing CIED surgery at our center between December 2015 and March 2017 and evaluate the feasibility and the clinical outcome of the PlasmaBlade™.

**Results:**

282 patients (mean age 70.7 ±  12.9 years; 65.6% male) were included, of which 119 (42.2%) underwent pacemaker implantation, 95 (33.7%) implantable cardioverter defibrillator implantation, and 68 (24.1%) received a generator replacement. At the time of the procedure, 55 patients (19.5%) were on dual antiplatelet therapy, and 109 (38.7%) patients were on oral anticoagulation (30.5% vitamin K antagonists, 8.2% novel oral anticoagulants). The overall perioperative complication rate was 3.9%. Device-pocket hematoma occurred in 9 patients (3.2%) requiring further surgery. No lead damage was seen within a follow-up of 6 months. One patient presented with device-pocket infection 2.9 months after implantation of a cardiac resynchronization therapy defibrillator requiring CIED system extraction.

**Conclusions:**

Replacing conventional electrocautery by PlasmaBlade™ for CIED procedures is feasible with a moderate rate of perioperative complications compared to the literature. Studies comparing the PlasmaBlade™ with conventional electrocautery are necessary to investigate whether PlasmaBlade™ offers an additional benefit over conventional electrocautery.

## Background

Due to the aging population, the number of cardiac implantable electronic device (CIED) implantations has increased over the last decades [1, 2]. Complications following CIED implantation are more frequent than generally acknowledged and range from 9.5 to 12.6% [3, 4] in large clinical trials and are associated with healthcare costs, patient discomfort, and increased morbidity and mortality. Early complications include pneumothorax, hematoma, infections, and lead dislocation [5–7]. One of the most common complications after device implantation is device-pocket hematoma (0.6–5.2%) [8, 9], especially in patients on oral anticoagulation. Generator replacements carry the additional risk of inadvertent lead damage, especially with the use of conventional electrocautery as the heating of the cautery tip may result in potential isolation defects [10].

The pulsed electron avalanche knife (PEAK) PlasmaBlade™ is a new low-thermal-injury electrosurgical device that uses brief, precise pulses of radiofrequency (RF) energy to cut and coagulate soft tissue. PEAK PlasmaBlade™ device allows touching the leads directly with the tip of the PlasmaBlade™ and to cut off all the tissue which is covering the leads. The precision of cutting and coagulation is controlled by the surgeon by adjusting the power level. The soft tissue dissection device of PEAK PlasmaBlade™ has been widely applied in ear, nose, and throat (ENT) procedures and is considered to provide scalpel-like cutting precision and bleeding control while producing less tissue injury and minimal scar formation [11]. Thermal injury depth, inflammatory response, and scar width appear to be reduced upon PlasmaBlade™ incisions in comparison to conventional electrocautery [12]. Thus, PlasmaBlade™ may provide significant advantages in wound healing. With respect to CIED surgery, the use of PlasmaBlade™ might accelerate wound healing and thereby reduce the rate of infections. In addition, there is no risk of lead damage during generator replacement compared to conventional electrocautery [13].

The aim of this study was to evaluate the feasibility and clinical outcome of the PEAK PlasmaBlade™ in patients undergoing all types of CIED surgery in our center.

## Methods

### Study design

From December 2015 to March 2017, patients undergoing pacemaker (PM), implantable cardioverter defibrillator (ICD) implantation, and generator replacements at our center were included in this retrospective observational study. The study was conducted in compliance to the Declaration of Helsinki, and the research protocol was approved by the Ethics Committee of the Medical Faculty of the University of Essen. According to the institutional review board and the retrospective design of the study, a written informed consent of the participants was not required.

### Procedures

CIED surgery was performed under conscious sedation with local anesthesia. All procedures were performed using the PEAK PlasmaBlade™ (Medtronic Inc., Minneapolis, MN, USA). We normally used PlasmaBlade™ CUT 5 and COAG 6 mode. Conventional electrocautery was not used. Novel oral anticoagulants (NOACs) were stopped at least 48 h before the procedure and were re-started 48 h after the procedure. In patients treated with vitamin K antagonist (VKA), VKA was continued with a target international normalized ratio (INR) between 2.0 and 3.0 on the day of surgery. In case of interruption of VKA, patients received bridging therapy with intravenous heparin. Heparin was reinitiated 24 h after the procedure and continued until a therapeutic INR was achieved. Intravenous heparin was preferred over low-molecular-weight heparin. Antiplatelet therapy was not stopped. All procedures were performed by a single experienced operator (E.K.). The preferred access for lead implantation was the vena cephalica. In case of a failed cephalic approach, a subclavian vein puncture was performed.

Complications were divided into infectious complications (superficial infections, pocket infections, and systemic infections requiring complete device extraction), lead damages during device replacement resulting in generator or lead malfunction requiring re-operation, device-pocket hematomas, as well as death from any cause within 30 days. Device-pocket hematoma was defined as hematoma requiring further surgery and resulting in prolongation of hospitalization. Prolongation of hospitalization was defined as extended hospitalization for at least 24 h after the index surgical procedure, primarily due to hematoma. Daily assessment of the device pocket and wound examination were performed until the patient was discharged from the hospital. Follow-up visits (including wound assessment) were scheduled 4 weeks, 3, and 6 months after the procedure regardless of the type of CIED surgery in our outpatient clinic.

### Statistical analysis

Baseline characteristics, procedure-related data, procedure-related complications, and follow-up date were recorded into a database. Statistical analyses were performed using SPSS statistical software version 23.0.02 (IBM SPSS Statistics for Mac, Version 23.0.02. Armonk, NY: IBM Corp). Continuous variables are expressed as mean ± standard deviation in case of normal distribution, as median and interquartile range in the case of other distribution. Categorical variables are summarized as counts and percentage. Since this was an exploratory analysis, no adjustment for multiple testing was applied.

## Results

### Patient and device characteristics

282 patients presented for CIED surgery between October 2015 and March 2017. 119 patients underwent PM implantation (27 single-chamber PMs, 79 dual-chamber PMs, 13 CRT-PMs), 95 patients ICD implantation (49 single-chamber ICDs, 15 dual-chamber ICD, 29 CRT-ICDs, 2 subcutaneous ICDs), and 68 patients received a generator replacement (27 pacemaker and 41 ICD generator replacements) (Table 1). Mean patient age was 70.7 ± 13.9 and 185 (66%) patients were male. Mean ejection fraction (EF) was 39 ± 13%. Dilative cardiomyopathy was present in 41 (15%) patients, ischemic cardiomyopathy in 40 (14%), and hypertrophic cardiomyopathy 3 (1%) patients. Forty-two (15%) patients had prior cardiac surgery. Nearly half of the patients (*n* = 135, 48%) had a history of atrial fibrillation. Therefore, a majority of the patients underwent CIED implantation on oral anticoagulation (Table 2). Eighty-six patients (30.5%) were treated with VKA, and 23 (8.2%) patients were on NOAC therapy. Among the NOACs, Apixaban was most frequently used followed by Rivaroxaban. Six patients underwent the procedure on triple anticoagulation, and 55 patients on dual antiplatelet therapy (DAPT). Baseline characteristics of the patients are listed in Table 3.

**Table 1:** List of CIED procedures

	*n* (%)
Pacemaker implantations	
Single chamber (VVI-PM)	27 (9.6)
Dual chamber (DDD-PM)	79 (28.0)
CRT-PM	13 (4.6)
ICD implantations	
Single chamber (VVI-ICD)	49 (17.4)
Dual chamber (DDD-ICD)	15 (5.3)
CRT-ICD	29 (10.3)
Subcutaneous ICD (S-ICD)	2 (0.7)
Generator replacements	68 (24.1)
PM generator replacement	27 (9.6)
ICD generator replacement	41 (14.5)

*PM* pacemaker, *ICD* implantable cardioverter defibrillator, *CRT* cardiac resynchronization therapy

**Table 2:** Peri-interventional anticoagulation

Anticoagulation	*n* (%)
VKA^a^	87 (30.9)
NOAC	15 (5.3)
Rivaroxaban	8 (2.8)
Edoxaban	2 (0.7)
Apixaban	15 (5.3)
Dabigatran	0 (0)
DAPT	55 (19.5)
Triple therapy	6 (2.1)

*VKA* vitamin K antagonist, *DAPT* dual antiplatelet therapy, *NOAC* novel oral anticoagulant

^a^Mean INR of patients on VKA at the time of the procedure was 1.51 ± 0.37

**Table 3:** Baseline demographic and clinical characteristics of the study population

Patients	*n* = 282
Demographics	
Age, years	71 ± 13
Male sex, *n* (%)	185 (65.6)
Body mass index (kg/m^2)^	27 ± 5
Medical history	
DCM, *n* (%)	41 (14.2)
ICM, *n* (%)	40 (14.2)
HCM, *n* (%)	3 (1)
CABG, *n* (%)	42 (14.9)
Ejection fraction (%)	39 ± 6
Atrial fibrillation, *n* (%)	135 (48.0)
Renal insufficiency, *n* (%)	79 (28.0)
Diabetes, *n* (%)	73 (25.9)

*DCM* dilatative cardiomyopathy, *ICM* ischemic cardiomyopathy, *HCM* hypertrophic cardiomyopathy, *CABG* coronary artery bypass graft surgery

### Procedural data

The mean overall procedure duration (time from first skin incision until the end of surgery) was 60.1 ± 49.3 min ranging from a minimum of 8 min for a simple PM generator replacement and a maximum of 320 min for a CRT implantation. The mean postoperative hospital stay of the patients treated with PlasmaBlade™ was 5.7 ± 4.0 days.

Complications occurred in 11 (3.9%) patients. Of these, 2 (1 pneumothorax and 1 atrial lead dislodgement) complications were seen within 24 h after the index procedure. Nine patients (3.2%) developed a significant device-pocket hematoma requiring surgical intervention. Of note all device-pocket hematomas developed 48 h after surgery. Seven of these patients (77.8%) were on oral anticoagulation (5 VKA, 2 NOACs), 2 patients were on DAPT. INR at the time of the index surgery was 1.62 ± 0.45. Only 1 patient underwent CIED surgery on uninterrupted VKA. No blood transfusion was needed. One pocket infection (0.35%) requiring complete device and lead extraction was seen. There were no damaged leads that had to be replaced after the index procedure or within the 6-month follow-up. One patient died within 30 days, which was not related to the CIED procedure. A detailed list of all complications is provided in Table 4.

**Table 4:** Procedure-related data

Total procedure time, min	60.1 ± 49.3 [8; 320]
Length of postoperative hospital stay, days	5.7 ± 4.0
Perioperative complications, *n* (%)	11 (3.9)
Pneumothorax, *n* (%)	1 (0.4)
Lead dislodgement, *n* (%)	1 (0.4)
Device system infection, *n* (%)	1
Superficial infection, *n* (%)	0
Significant device-pocket hematoma, *n* (%)	9 (3.2)
Lead damage, *n* (%)	0 (0)

## Discussion

PlasmaBlade™ is a promising novel surgical tool that provides atraumatic, scalpel-like cutting precision and electrosurgical-like hemostasis, resulting in minimal bleeding, tissue injury, and scar formation. Acute thermal injury depth was reduced by 74% [12]. PlasmaBlade™ incisions demonstrated reduced inflammatory response and scar width in healing skin compared with conventional electrocautery or scissors. Within the context of CIED surgery, PlasmaBlade™ might therefore provide clinically meaningful advantages over conventional electrocautery by accelerating the healing process, reducing the risk of infection and avoiding inadvertent lead damage. This again may lower overall hospital costs compared to conventional techniques.

There are currently data supporting the use of PlasmaBlade™ in patients undergoing PM or ICD generator replacement [13]. Generator replacement is associated with the risk of developing pocket hematoma and/or infection and inadvertent damage to the leads, especially when leads have to be freed from surrounding fibrous tissue using conventional electrocautery or scissors. No studies exist evaluating the use of the PlasmaBlade™ instead of conventional electrocautery for all types of CIED procedures. Our data are the first to demonstrate that the PlasmaBlade™ can be used instead of conventional electrocautery for de novo CIED implantations and generator replacements with comparable procedure duration and a low risk of wound infections within a follow-up of 6 months compared to the literature.

The overall perioperative complication rate in our population was 3.9% which was mainly attributable to a considerable amount of device-pocket hematomas (3.2%). Considering the fact that 38.7% of the patients were on oral anticoagulation and 19.5% on DAPT, this is most likely a result of the anticoagulation regime, but not a problem associated with the general use of the PlasmaBlade™. Furthermore, the mean INR of patients on VKA at the time of the procedure was 1.51 ± 0.37 [0.99; 2.75] suggesting that only a small amount of patient underwent CIED surgery on uninterrupted VKA. It is most likely that interruption of VKA was contributing to the higher rate of hematomas compared to the literature according to the results of the BRUISE CONTROL study [14] and was resulting in a prolonged postoperative hospital stay [15, 16].

Despite the above-mentioned advantages of the PlasmaBlade™, the acquisition costs of the PlasmaBlade™ are distinctly higher than those of a conventional electrocautery unit. Further data demonstrating a reduction in the overall complication rate, procedure time, and length of hospital stay translating into cost savings are necessary until PlasmaBlade™ might replace the conventional electrocautery unit.

### Limitations

The major limitation of our study is that it is a non-randomized, retrospective single-center study. Furthermore, as PlasmaBlade™ was used in all patients, there was no control group, in which conventional electrocautery as the standard of care was applied. We were indeed able to demonstrate the feasibility and safety of replacing conventional electrocautery by PlasmaBlade™ in a “real-world” setting in all types of CIED procedures, but it remains to be seen whether PlasmaBlade™ might be superior to conventional electrocautery.

## Conclusions

The use of PEAK PlasmaBlade™ instead of conventional electrocautery during de novo PM and ICD implantations and generator replacements seems to be feasible and is associated with a moderate risk of complications. Further studies comparing PlasmaBlade™ and conventional electrocautery are warranted to evaluate whether PlasmaBlade™ is superior to conventional electrocautery for CIED procedures.

## References

[1] Wilkoff BL, Auricchio A, Brugada J, Cowie M, Ellenbogen KA, Gillis AM, Hayes DL (2008). HRS/EHRA expert consensus on the monitoring of cardiovascular implantable electronic devices (CIEDs): description of techniques, indications, personnel, frequency and ethical considerations. Heart Rhythm.

[2] Uslan DZ, Tleyjeh IM, Baddour LM, Friedman PA, Jenkins SM, StSauver JL, Hayes DL (2008). Temporal trends in permanent pacemaker implantation: a population-based study. Am Heart J.

[3] Udo EO, Zuithoff NP, van Hemel NM, de Cock CC, Hendriks T, Doevendans PA, Moons KG (2012). Incidence and predictors of short- and long-term complications in pacemaker therapy: the FOLLOWPACE study. Heart Rhythm.

[4] Kirkfeldt RE, Johansen JB, Nohr EA, Jorgensen OD, Nielsen JC (2014). Complications after cardiac implantable electronic device implantations: an analysis of a complete, nationwide cohort in Denmark. Eur Heart J.

[5] Ellenbogen KA, Hellkamp AS, Wilkoff BL, Camunas JL, Love JC, Hadjus TA, Lee KL, Lamas GA (2003). Complications arising after implantation of DDD pacemakers: the MOST experience. Am J Cardiol.

[6] van Eck JW, van Hemel NM, Zuithof P, van Asseldonk JP, Voskuil TL, Grobbee DE, Moons KG (2007). Incidence and predictors of in-hospital events after first implantation of pacemakers. Europace.

[7] Nowak B, Tasche K, Barnewold L, Heller G, Schmidt B, Bordignon S, Chun KR (2015). Association between hospital procedure volume and early complications after pacemaker implantation: results from a large, unselected, contemporary cohort of the German nationwide obligatory external quality assurance programme. Europace.

[8] Kiviniemi MS, Pirnes MA, Eranen HJ, Kettunen RV, Hartikainen JE (1999). Complications related to permanent pacemaker therapy. Pacing Clin Electrophysiol.

[9] Wiegand UK, LeJeune D, Boguschewski F, Bonnemeier H, Eberhardt F, Schunkert H, Bode F (2004). Pocket hematoma after pacemaker or implantable cardioverter defibrillator surgery: influence of patient morbidity, operation strategy, and perioperative antiplatelet/anticoagulation therapy. Chest.

[10] Poole JE, Gleva MJ, Mela T, Chung MK, Uslan DZ, Borge R, Gottipaty V (2010). Complication rates associated with pacemaker or implantable cardioverter-defibrillator generator replacements and upgrade procedures: results from the REPLACE registry. Circulation.

[11] Loh SA, Carlson GA, Chang EI, Huang E, Palanker D, Gurtner GC (2009). Comparative healing of surgical incisions created by the PEAK PlasmaBlade, conventional electrosurgery, and a scalpel. Plast Reconstr Surg.

[12] Ruidiaz ME, Messmer D, Atmodjo DY, Vose JG, Huang EJ, Kummel AC, Rosenberg HL (2011). Comparative healing of human cutaneous surgical incisions created by the PEAK PlasmaBlade, conventional electrosurgery, and a standard scalpel. Plast Reconstr Surg.

[13] Kypta A, Blessberger H, Saleh K, Hönig S, Kammler J, Steinwender C (2015). An electrical plasma surgery tool for device replacement–retrospective evaluation of complications and economic evaluation of costs and resource use. Pacing Clin Electrophysiol.

[14] Proietti R, Birnie DH, Healey JS, Verma A, Essebag V (2013). Continued oral anticoagulation during cardiac pacing: the BRUISE CONTROL study. G Ital Cardiol (Rome).

[15] Markewitz A (2015). Annual Report 2013 of the German Cardiac Pacemaker and Defibrillator Register—part 1: pacemaker. Pacemaker and AQUA Institute for Applied Quality Improvement and Research in Health Care GmbH workgroup. Herzschrittmacherther Elektrophysiol..

[16] Markewitz A (2015). Annual Report 2013 of the German Cardiac Pacemaker and Defibrillator Register—part 2: implantable cardioverter-defibrillators. Pacemaker and AQUA Institute for Applied Quality Improvement and Research in Health Care GmbH workgroup. Herzschrittmacherther Elektrophysiol..

